# *Living Proof*: centering recovery narratives in public health communication

**DOI:** 10.3389/fpubh.2026.1861243

**Published:** 2026-07-10

**Authors:** Jeffrey K. Hom, Eileen Loughran, Malaika Fraley, Deirdre Hussey, Michael Alvarez, Jean Bruno, Juliana Gurrola-Nuno, Jen Jeffries, Dominique McDowell, Gregory McDowell, Shavonne Wong, Christine Soran, Hillary Kunins

**Affiliations:** 1 San Francisco Department of Public Health, San Francisco, CA, United States; 2 Personal Capacity, San Francisco, CA, United States

**Keywords:** addiction, mass media, media campaigns, public health, recovery, stigma, substance use disorders

## Abstract

In the United States, stigma about substance use, addiction, and treatment affects people’s awareness of and willingness to seek effective services. Additionally, reducing stigma is vital to decrease overdose deaths and implement compassionate policy and practices. To counter prevailing, judgmental attitudes about substance use, the San Francisco Department of Public Health launched the *Living Proof* campaign in November 2024. The campaign featured testimonials from San Francisco residents with lived experience, who shared their journeys as evidence that treatment works and that recovery is possible. Through January 2026, the campaign generated an estimated 210 million impressions across billboards, transit shelters, radio, local newspapers, and on social media, exceeding projected impressions. Public polling found high rates of message clarity, with 28% of viewers recalling the campaign unaided, 52% endorsing greater sympathy toward people with a substance use disorder, and 44% reporting that they were likely to take action after seeing the campaign. This community case study offers practice-based lessons for other jurisdictions considering their own campaign and highlights the critical importance of valuing lived experience as expertise.

## Introduction

Despite recent gains, nearly 70,000 people continue to die annually of unintentional drug overdoses in the United States (1). Furthermore, profound disparities exist among overdose decedents, with disproportionately high rates among Black individuals and American Indian and Alaskan Native individuals and rapidly increasing rates among youth (2, 3). In the context of this enduring yet evolving crisis, it is important that public health agencies refine how they communicate about substance use, a highly stigmatized topic. Drug-related stigma limits access to services, slows needed policy reform, and undermines empathy (4).

Evidence from the communications literature broadly shows that the framing of health issues influences how they are perceived and what policies and interventions are possible (5–9). Substance use has been historically framed in the United States as largely an issue of moral failing or one that requires a criminal-legal response (10, 11). These portrayals have been reinforced through the media’s depictions of substance use, especially toward communities of color, but also through past public health campaigns (12–14). The “Just Say No” campaign of the 1980s, for example, instructed youth to resist peer pressure, but maintained the perception of drug use as a moral failing by framing it as a personal choice (15).

Although the legacies of “Just Say No”, the Drug Abuse Resistance Education (D. A. R. E.) program, and the “this is your brain on drugs” campaign have endured, research shows that fear-based depictions of substance use are generally less effective at changing behaviors and may contribute to the stigma faced by people who use drugs (16–18). A growing body of evidence suggests that narratives of recovery, rather than suffering or crisis, are more effective in reducing negative perceptions (19–21). In Scotland, the “How to Save a Life” campaign used a message of empowerment to increase awareness and uptake of naloxone, demonstrating that asset-based framing can drive concrete action on a drug-related issue (22). However, evidence on recovery-focused campaigns in the United States remains limited.

Recovery-focused narratives work in part by activating humanizing frames rather than stigmatizing ones: viewers are invited to see people who use substances as full individuals navigating a health condition, not as social problems. In contrast to crisis-focused messaging, which may reinforce fear or hopelessness, recovery narratives foreground agency, opportunity, and community belonging (23).

Mass media campaigns are a well-established public health tool for reaching large populations with health messages, shaping social norms, and shifting attitudes that influence behavior (24). They have been used in efforts to reduce stigma, where the goal is not individual behavior change alone but a broader shift in how communities perceive a health condition (20, 25). Campaigns that achieve wide reach can normalize help-seeking, reduce the social penalties associated with a stigmatized condition, and create conditions under which policy change becomes more politically feasible (26).

Using this evidence, San Francisco Department of Public Health launched *Living Proof*, a mass media campaign that portrayed how recovery is possible and that treatment works. This community case study shares how a recovery-focused narrative was operationalized, the rationale for centering the lived experience of people in recovery, and practice-based lessons the campaign may offer to others in public health communications.

## Materials and methods

Qualitative data presented in this article were derived from recorded, semi-structured interviews conducted with *Living Proof* campaign participants as part of the campaign’s evaluation. Interviews were conducted by health department staff and professionally transcribed for analysis. Themes were identified through review of transcripts by the authorship team. Quantitative data were obtained from a public opinion survey conducted by an independent research firm. The firm administered a probability-based online survey, applying quotas based on Census data to ensure the sample reflected the demographic and geographic diversity of San Francisco adults (*n* = 507). This article is a collaborative effort by health department staff involved in the campaign’s development and individuals who participated in *Living Proof* as featured community members. This unique authorship structure accounts for the first-person, practice-based perspective reflected throughout.

### Designing “*Living Proof*” and centering lived experience

With *Living Proof*, a campaign that was first developed in the New York City Department of Health and Mental Hygiene in 2017, the San Francisco Department of Public Health sought to implement strength-based messaging through the use of visuals and testimonials.

The campaign was developed and implemented by a core team within the San Francisco Department of Public Health’s communications division and those featured in the campaign, working in close partnership with a creative design consulting company, a production firm responsible for photography and videography, and media buying agencies that managed placement across print and digital channels. Planning began approximately 15 months prior to the November 2024 launch.

The campaign’s objectives were to (1) increase awareness that treatment for substance use disorders is available and that recovery is possible, (2) increase awareness that medications for opioid use disorder are highly effective and available, (3) direct people with substance use disorders and/or their loved ones to treatment resources, and (4) reduce drug-related stigma through the use of humanizing profiles. The campaign participants themselves reflected on these goals and being part of *Living Proof*:

*One of my major driving forces to be a part of the campaign was my own experience of not having any outreach done to me or any familiarity with any sort of resources. Even when I did go and get treated, there still wasn't a lot of education and there were a lot of missed opportunities. Being also a Black American in the Tenderloin and in San Francisco, being born and raised, I thought it was also very important to have representation.*

*I think that it helped people know that there's help out there, and there's different routes to it, whether it's detox, treatment, [medications for addiction treatment], whatever that looks like. I feel like when they see us who are actually in recovery and a part of that, it makes it more realistic and more attainable.*

Participants were identified through existing relationships between health department staff and community-based organizations serving people with substance use disorders, as well as through referrals from trusted providers and peer networks. Individuals were approached if they felt stable in their recovery, were willing to share their story publicly, and reflected the demographic diversity the campaign sought to represent. Eleven individuals participated across the campaign’s three waves.

Individuals with lived experience in *Living Proof* were not solely “featured” in the campaign, but active participants in shaping how and where their stories were told (27). Communications staff gave guidance about this being an aspirational campaign but were not prescriptive in specifically what elements of participants’ stories should be shared. Creative decisions on the following were made by those in the campaign, with the support of the health department:

- How they appeared and with whom – by themselves or with family members
- Where they were filmed – where was safe, meaningful, and personal to them
- What elements of their story they shared on camera – how did they want to present themselves

*Living Proof* was also developed with inclusion in mind, ensuring the campaign reflected the diversity of San Francisco. Substance use, addiction, and recovery span all ages, races and ethnicities, languages, sexual orientations, and gender identities (28). It was felt essential that the primary audience – San Francisco residents who use substances – relate to those in the campaign. At the same time, care was taken to avoid tokenism, which would have been antithetical to the spirit and key message of the campaign. This meant supporting campaign participants to share their stories with authenticity and dignity.

While *Living Proof* was meant to be humanizing and inspirational, that it was also about substance use was always front of mind (29). It was critical that those participating in the campaign, whose faces would be on billboards, bus shelters, and public transit in their communities, as well as on social media, understood the implications of their participation. San Francisco is a geographically small city and visibility of campaign was likely to be high on the city’s major thoroughfares. Some participants described understandable concern about being labeled as someone who used drugs, no matter how well they were doing now.

*The only thing that I feared was parents from my daughter's school recognizing us and not knowing that I had that past. At the end of it, I was just like, I shouldn't care what they think of me. That's not who I am today. I think that was just the only fears, other people from my daughter's classroom or their parents would see me, teachers and stuff, and judge me, or something like that. It never happened.*

Obtaining informed consent was consequently not just a formality, but relational. Time and effort were taken with each participant in the campaign to understand their motivations for participating, and to ensure comfort and safety throughout.

*I was able to trust the process and trust the project, because I think trust is really important. We are putting ourselves out there. We're putting our personal lives out there. I really felt safe. The way you guys did it was really safe and comfortable.*

All ultimately expressed a strong desire to be part of the campaign, recognizing that by sharing their story and success they could perhaps help others who were in their situation.

*I just want people to know that even with all those barriers and those challenges, I overcame something, and that there are people who are going through the same thing. I want them to know that they can overcome it.*

*I lost my belief in myself. A lot of times, I felt like my life was going nowhere. To see that, to see me up [on a billboard] and showing how far I've come, it's great. If I would have seen something like that when I was just getting into my addiction, then I could have seen that there was hope to get out of it.*

People were compensated for their participation, and the San Francisco Department of Public Health has maintained relationships with those featured, seeking their ongoing partnership if they choose. It is essential that partnerships with people with lived experience not be a “one and done”, but instead an enduring, respectful relationship that involves going beyond one or two campaigns to broader decisions, events, education, and policies (30).

*I think it's great that we were compensated, but I think that when community members, people with lived experience are invited into a campaign like this, that we are compensated what we are worth. I think that that's because there's an ask. It’s not a one-time ask, because although this is your work, this is for the rest of our lives.*

*We should always be at the table when there's new policies, new promotions, new procedures coming out. We got to always think about, it’s always got to be equitable. It’s always got to be for everybody, you know what I'm saying? I think you find somebody with that mindset, with lived experience, I think they should always be at the table. It's very important for their message to get across when things are coming out to help the population that they're in.*

### Campaign channels and community reach

**Figure 1:**
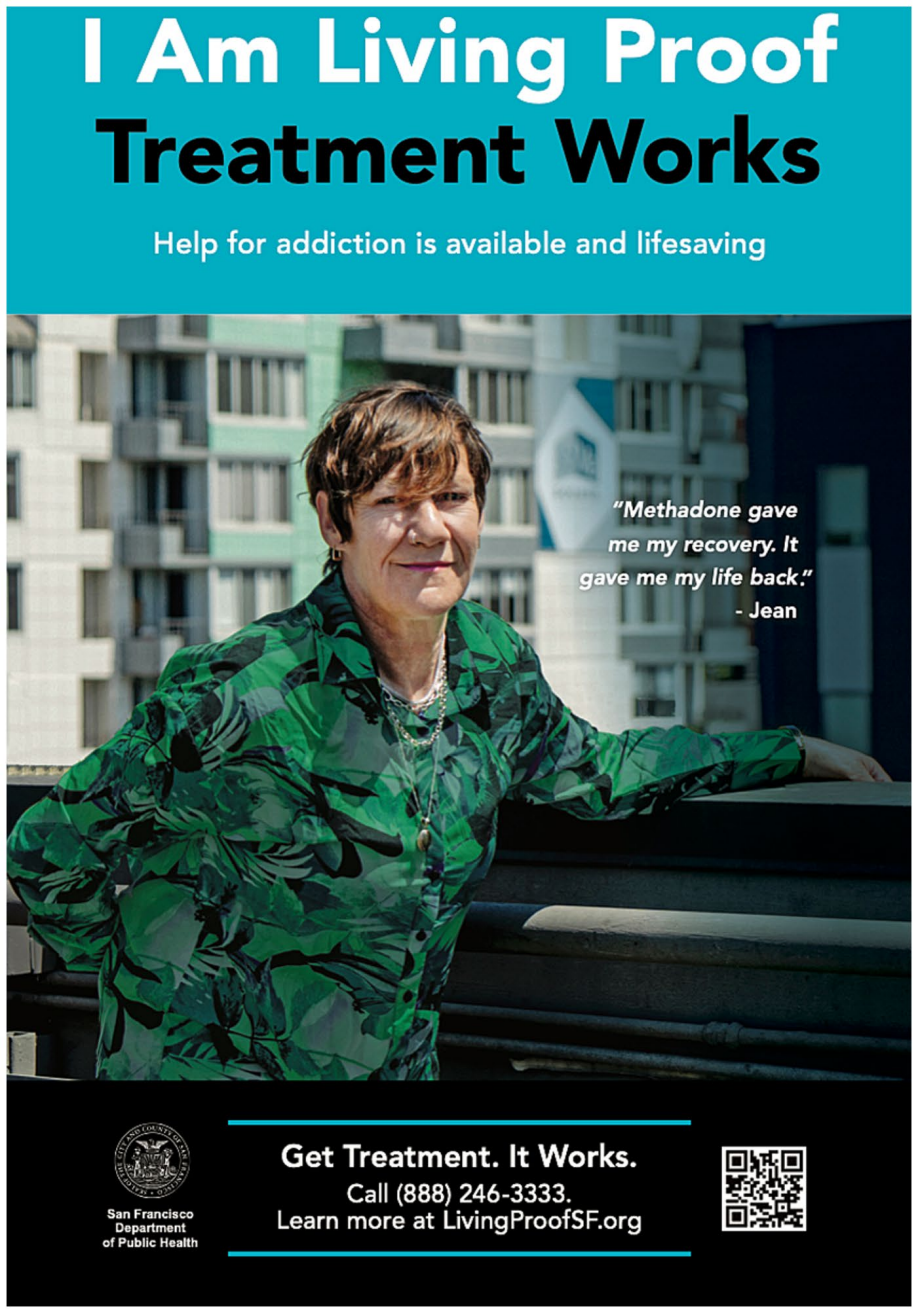
Campaign poster for *Living Proof*, San Francisco, CA, USA, August 2025. San Francisco Department of Public Health. Reproduced with permission from “Living Proof Precident Posters” by the San Francisco Department of Public Health (https://www.sf.gov/departments--department-public-health).

The primary audience was people with substance use disorders and/or their family members in San Francisco. The secondary audience was the San Francisco public. The campaign was implemented in three waves between November 2024 and January 2026, each featuring a refreshed set of creative assets (Figure 1). Ads placed in print media (including billboards, transit infrastructure, local newspapers) were updated at the start of each wave to sustain audience engagement and reduce ad fatigue. Organic social media via the health department’s profiles was maintained between waves, whereas paid social media advertisements were purchased at the start of each wave. The campaign also involved a partnership with a ride-share company for in-car tablet placements and coordination with community-based organizations for poster distribution in clinical and social service settings. Most of the campaign’s costs covered media placement across print and digital channels; creative production and staff time represented a smaller proportion of the total expenditure.

### From story to strategy: embedding narrative in the public health infrastructure

Like most public health campaigns, *Living Proof* included a call to action. The campaign was about changing minds, but it was also meant to communicate about the health department’s systematic approach to treating substance use disorders and preventing overdoses. Considerable effort and investments have been made to expand the options and capacity of San Francisco’s treatment system, including an increase of more than 100 residential treatment beds in the past two years (31). This campaign intended to promote availability of new and existing resources available in the city.

*Now that I'm working with all these families and helping them, it's definitely nice to look back and see, "Hey, look at all these families that found treatment and are able to find a good plan and find housing.” The support is here in San Francisco if you want it. There're so many things that can support you.*

*I think it's important for people that are reading the article or seeing the videos, to really understand that there is a way to move out of this, and that there really are a lot of organizations, especially in San Francisco, that help. There's just so many organizations, and the people that really work there really do care. It's important for people to realize that, because it can be really scary just walking in the door at a lot of places, that stigma is there, that fear is there, that shame is there. It's important for people to know that the people that work in these organizations really do care.*

*Living Proof* specifically launched alongside the San Francisco Department of Public Health’s expansion of its night navigator and telehealth buprenorphine program (32). By messaging hope and possibility through real-life recovery stories, the *Living Proof* campaign was intended to build trust in the system, promote the belief that treatment works, and encourage engagement with city services. Furthermore, the campaign’s humanizing tone also made an impact within the health department. Upon seeing *Living Proof* and hearing positive feedback from partners and the public, staff have reported a greater awareness about the issue and felt a deeper appreciation for lived experience as expertise. Anecdotally it also fostered within the health department a renewed, shared commitment to supporting low-barrier treatment options for the safety net population in San Francisco.

## Results

*Living Proof* was visible to thousands of people in San Francisco, while the evaluation indicated that the messages resonated and increased viewers’ self-reported motivation to take action. The campaign is estimated to have garnered over 210 million impressions through January 2026 via billboards, transit shelters, radio, local newspapers, and social media, exceeding projected impressions. Weekly traffic to the city’s behavioral health website nearly doubled following the campaign’s launch – rising from an average of 1,263 visits per week in the 6 months prior to the third wave’s launch (March–August 2025) to 2,362 visits per week during the months that all campaign products were fielded (August–November 2025). Table 1 summarizes additional campaign metrics by channel.

**Table 1:** Summary of *Living Proof* campaign metrics, San Francisco, California, USA, 2024–2026.

Channel	Metric	Result (through January 2026)
Print (billboards, transit shelters, buses)	Estimated impressions	181,599,150
Social media (Meta, Simplifi, YouTube)	Estimated impressions	16,774,479
Local publishers	Estimated impressions	2,614,010
Radio ads	Estimated impressions	9,660,860
All channels combined	Total estimated impressions	210,648,499
Social media (Meta)	Click-through-rate	1.4%
Social media (Meta, Simplifi, YouTube)	CPM	$3
Behavioral health website	Avg. weekly visits, pre-third wave launch (March–August 2025)*	1,263
Behavioral health website	Avg. weekly visits, post-third wave launch (August–November 2025)	2,362
Behavioral health website	Change in weekly visits	87%
Public opinion survey	Sample size	507
Public opinion survey	Unaided campaign recall	28%
Public opinion survey	Aided campaign recall	37%
Public opinion survey	Reported ad caught their attention	54%
Public opinion survey	Reported greater sympathy toward people with SUD	52%
Public opinion survey	Reported likely taking action after viewing	44%
Earned media	News stories generated	6

*Pre-March 2025 data unavailable due to website migration.

In addition to tracking impressions, the health department invested in polling to understand how the public perceived the campaign: toward the end of the campaign’s third wave, a sample of 507 San Franciscans reflecting the city’s diversity were surveyed. Twenty-eight percent of respondents recalled seeing *Living Proof* unaided, well above the unaided recall of three fictious campaign names serving as controls (3–14%). Approximately 37% of the respondents recalled seeing the printed or online ads when shown an image from the campaign (aided recall). Among all survey respondents, 73% reported easily understanding what the ad was about (treatment and recovery from drug addiction) and 54% reported that it caught their attention. Fifty-two percent reported feeling more sympathy toward people with a substance use disorder because of the ad. Nearly 45% of respondents said they would likely take action after viewing the ad, such as visiting the department’s website or sharing the information with family or friends. These results suggest that the campaign reached its intended audience, reduced stigma, and helped motivate action.

Beyond indicators of visibility and resonance, the campaign generated meaningful reflections from participants about their experience, motivations, and hopes for impact. These perspectives, while not formal outcome measures, offer insight into the campaign’s significance for those who made it possible and illustrate the human dimensions of recovery-focused storytelling. Campaign participants were impressed by the campaign’s scale and how it reached so many of friends, family members, and acquaintances. This led to positive feedback and supportive messages.

*All of my friends that are seeing the posters, the billboards, and stuff, I'm getting calls from people that I haven't talked to in 15, 20 years. That was really cool to see that the message, especially in Bayview, my old neighborhood, a lot of people reached out to me. They're searching out my phone number. Like, "Hey, man. We've seen you. We're so proud of you."*

*Our friends kept texting us and taking pictures and being like, “we see you guys”. That was good. Just them recognizing that we've overcome something that really had a strong hold on us. I was super excited and I felt good. I just think that they were like, “you guys are like killing it. You guys are helping and helping other people”. Even with our friends, similar things, so proud of how far we've come and just the challenges that we've overcome. I think that they recognize that.*

Importantly, the campaign helped the public see people with substance use disorders differently and provided an opportunity for education and engagement:

*I think it's super important for people who have never experienced substance use or anything like that, just to educate them a little bit on the fact that everybody is a human being, and we're not just people on the street, or drug addicts. We're people who have used substances. We're not just a number, or a statistic, or a problem, or whatever they want to look at it as. I feel like this campaign really put it in another light for all kinds of folks, whether you're on the substance use side, or the public health side, or just a part of the community. I just think it's really beneficial for everybody that gets to see it.*

*A lot of people have come up to me, and have seen my picture, and said "Wow, that's so great," and wanting to know how you did that. People in my building, especially where I live, coming home from work or something and seeing my picture, and they're like, "Wow, that's Jean." They come and talk to me, and I get to share about my experience with drug addiction, and getting out of it, and how I got out of it, and how do I stay out of it, and how it's affected my life, and that's been really the best thing for me.*

Perhaps most critically is that campaign participants were able to communicate directly to people who have substance use disorders and may be struggling and/or seeking help, who were the primary audience of the campaign. Consistent with the goals of recovery-focused narratives, *Living Proof* was intended to project hope and instill a sense of possibility, including in San Francisco residents who have not necessarily sought services.

*We threw a big seed into the community because we were all over the community. We dropped seeds everywhere. We planted stuff in the community and there's going to be some growth out of it. It's just the way it works. Seeds going to come out, and we just got to sit back and water it. I think we just be patient and let it all unfold the way it's going to unfold. I think we're saving a lot of people without even knowing we're saving them, just with the conversation. Just the conversations, just for a person to see. Faith comes from seeing, right? I think people got to see the proof. I'm talking about, we got to attract things. What we put out is what we get back.*

*I have a client who is taking medication for his recovery, and he sees me as a case manager, and he sees me taking the same medication and he doesn't have to feel like bad or guilt about it or shame. He can see people thriving and being on this medication, or people thriving and going through the same journey that he went through. When you're battling addiction and trying to recover, there can be all these little signs that point you that say “I need to go back to using”, but then you see success stories or other people who have been through similar stories like you and then you can see, "Okay, this is doable. It's not impossible."*

Reflecting on their contributions to *Living Proof*, campaign participants described their reactions, hopes, and the importance of valuing lived experience. Additional meaningful quotes about these build upon the preceding quotes and are included in Table 2.

**Table 2:** Additional representative quotes from *Living Proof* participants, San Francisco, California, USA, August 2025.

Topic	Representative quotes
On deciding to participate	*What made me participate in the Living Proof campaign was I am living proof. I think that was a big factor that let me know that it can be done. It’s not about promotion, it’s about attraction. We want to attract people to it that we know.* *I know that there’s a good 200 people that know me as an addict in San Francisco, an active user. My hope was to reach them, and they’d look at my picture and go, “I know her,” and read it, and go, “Hey, if she can do it, maybe I can do it. Maybe call this number, and I can get some help, because she was really hopeless.”*
On receiving feedback from others	*I know the people that know me personally, like I said, it turned their heads, and it still does, and it’s a great feeling to have a lot of people turn their head and go, “I know her.”* *I’m pretty visible in the community. I remember this one man that walked up to me, I think we were Facebook friends. He said, your face being up there on that poster said everything. Then he shared his journey with me. Then another time where I walked into a nonprofit organization with young people and I walked in, this young lady says, “You’re famous.”*
On being in the campaign	*[Being part of the campaign] made me more determined about my recovery. I’m coming up on three years, and I’m happy about that. I’m always my happiest in my life is when I’m in recovery. I’m not going back, there’s nothing there. It really has made my life just such a better place to be. My recovery is everything to me. It’s number one. Without my recovery, I would not have anything. I know that. I’m not willing to sacrifice my life, my relationships with my family, my children, with my friends, I’m not willing to do it. I’m not willing to go back. It just gets better. It just keeps getting better and better.*
On the campaign’s message	*I really enjoy the fact that it’s out there, and it shows people that there is a way to come out of that, and to move forward at whatever that looks like for people.* *I think that that campaign just gave some people a moment to just think about where they want to go next or what they want to do next, and if this was a chance that maybe they could try to start their recovery. For someone to go into a recovery, I think they need a little seedling of belief or a little seedling of hope. If that seedling is there, when they see something, for example, that poster or something, then that seed has a chance to sprout into like, “Okay, I’m going to go for it.”*
On the opportunity to educate others	*I was at an event where there were fire paramedics and first responders that often come to the Tenderloin. I shared my story with them and that I was part of this campaign and how important it was for them and doing their job, to actually see people who have had a story, a journey like mine, who was definitely a very sick addict, who would wake up to paramedics and in the emergency room, for them to see someone on the other side, to come on the other side because I do not necessarily look like what I’ve been through. To be able to communicate and articulate my story and then them to have real life firsthand experience with me in person and the work that I do.* *I just think that some people have lost hope and are just like, “these people are always going to be out here.” For them to see other people who have overcome it, I would hope that they took it positively and said, “okay, maybe some people can change and get over this.”*
On the importance of including lived experience	*I hope that there is more support for people like us and people who have lived experience, just to have our voices heard and where they most count.* *It’s important to have people who have been in the experience, lived experience, and use their knowledge to see what works and try to help out the homeless and the people who are really struggling.* *Just taking account from people who have battled addiction and have come out on top, maybe just learning from them or hiring more people like that, hiring more community health workers. That’s how I started.*
On suggestions for next steps	*I think that if you got the people that were a part of [the campaign] to go out into the street and hand out flyers or pamphlets around different types of resources, and get out there and talk to people, I think that would make a huge difference.* *Do not let this campaign end. Do not let this campaign die. It’s too important. There are so many other people who are doing well, they should be included too. The message needs to keep being out there.*

## Discussion

The San Francisco Department of Public Health treated *Living Proof* as an awareness campaign, but also one with a clear call to action. Centered around the testimonials of people who are “living proof” that treatment works and recovery is possible, the campaign was about shaping public understanding of substance use disorder as treatable, just as much as it was about promoting city services. The San Francisco Department of Public Health is relaunching *Living Proof* in summer 2026 to promote recall and capitalize on the favorable responses from polled city residents.

Other jurisdictions considering how to best frame their work can benefit by understanding what messaging will most resonate with their residents by partnering with people with lived experience, in a genuine, respectful, and lasting way.

### Implications for public health practice

The experience of *Living Proof* offers several important implications for other jurisdictions fielding public health campaigns:

1. Invest early and intentionally: Communications should be viewed as having a strategic function, not merely reactive messaging. Co-creation with community partners, prioritized from the outset, was essential to ensuring *Living Proof’s* authenticity and reach. The campaign generated over 210 million impressions and nearly doubled visits to the city’s behavioral health website – measurable returns on this investment. A budget should be allocated to support co-creation from the start.
2. Know your environment: Geography, political dynamics, and local attitudes should all inform a public messaging campaign. *Living Proof* was designed with San Francisco’s specific communities and media landscape in mind, including targeted placement in the Bayview and Tenderloin neighborhoods. Campaign participants reflected the city’s diversity, helping the primary audience – people who use substances – see themselves in the campaign. Investments in community input can improve a campaign and help key messages land with the intended audience.
3. Treat lived experience as expertise: Lived experience should be valued for leadership, not merely for input. In *Living Proof*, participants shaped not just the content but the form of their storytelling. Their ongoing desire to extend the campaign (“Don’t let this campaign die”) reflects the depth of their investment and argues for sustained partnerships – not only in campaigns, but in planning and policy discussions as well. Jurisdictions should prioritize involving community members in authentic, enduring ways, and compensate them appropriately.
4. Align the message with the system of services: Public health storytelling is most powerful when paired with accessible, high-quality services. *Living Proof* launched alongside the expansion of SFDPH’s telehealth buprenorphine program and an increase of over 100 residential treatment beds. Among those surveyed, 44% reported they would likely take action after viewing the campaign, a finding that is only meaningful if services are in place to meet the expectations established by the campaign.

There are several limitations to this work. First, this campaign was fielded starting in 2024 in one city in California, USA, and the prevailing attitudes that shaped and supported *Living Proof* may not be generalizable elsewhere at this time. Second, while polling revealed an *intent* to take action as a result of seeing the campaign, outcomes such as treatment enrollment could not be assessed which would have strengthened the evaluation. Third, while *Living Proof* was reported on positively by local news outlets, no formal content or sentiment analysis of this coverage was conducted, which would have provided a more rigorous assessment of how the campaign was perceived. Fourth, the cost to field large media campaigns is substantial and smaller health departments may face greater barriers to replication. However, strategic use of earned media may help increase the visibility of smaller campaigns when less funding is available.

Notwithstanding these limitations, the lessons from *Living Proof* are broadly applicable to health departments and public health communicators. Recovery-focused, narrative-centered campaigns that authentically center lived experience represent a replicable, evidence-aligned approach to stigma reduction and behavior change and are needed in the context of the ongoing overdose crisis. The campaign’s results across multiple indicators of reach, resonance, and motivation to act provide a real-world proof of concept for this approach and offer practical lessons for other jurisdictions to consider.

## Data Availability

The raw data supporting the conclusions of this article will be made available by the authors, without undue reservation.
